# Rush Medical College

**Published:** 1855-07

**Authors:** 


*Rush Medical College:*

It will be seen by the Annual Announcement of this College, which is issued with the present number of the Journal, that some changes and additions have been made in its faculty, and some very important improvements in regard to a College edifice.

The chair of Physiology and Pathology, vacant during the last three years, has been filled by the appointment of Wm B. Herrick, M. D., who has been for many years Professor of Anatomy and Physiology in the same school.

The chair of Anatomy, made vacant by the transfer of Professor Herrick, has been filled by the appointment of J. W. Freer, of this city, who has filled the place of Demonstrator and Lecturer on Descriptive Anatomy during the last four or five years. The chair of Materia Medica, made vacant by the resignation of Professor McLean, has been filled by the appointment of Dr. H. A. Johnson, of this city, who gave the course of Lectures on Physiogy during the session of 1853–4, with much satisfaction to all parties. To fill the office of Demonstrator and Lecturer on Comparative Anatomy, the faculty have been fortunate in securing the appointment of Prof. E. Andrews, lately connected in the same capacity with the Medical Department of the University of Michigan. These are excellent appointments, and will doubtless prove equally satisfactory to the College and the profession generally. The old College building has been taken down, and the work is making rapid progress towards the completion of a new edifice, with all those improvements and accommodations which the Institution requires and modern architecture affords.—Though the past history of the Rush Medical College has been one of uniform prosperity, yet the improvement now made will be followed by a more extended patronage, and a still higher reputation.

Prof. John McLean, tendered his resignation soon after the close of the session of 1854–5. The following Preamble and Resolution reported by a committee of the faculty appointed for that purpose, afford but a just tribute to his moral worth and professional attainments:

WHEREAS, John McLean, M. D., of Jackson, Michigan, has been connected with the faculty of Rush Medical College, first in the capacity of Prof. of Practice, and subsequently filling the chair of Materia Medica, from the foundation of the Institution; and whereas, he has this day signified his desire to be relieved from the further responsibility of the chair which he has filled so long and successfully by sending in his resignation of said place, Therefore,

*Resolved*, That the Faculty of Rush Medical College, in parting with their late colleague, Prof. John McLean, with whom they have been associated for several years, take pleasure in bearing testimony to his high moral qualities, his uniformly, kind and gentlemanly deportment, and his faithful discharge of the duties imposed on him as teacher of an important department of Medical Science.

